# Health for Children

**Published:** 1856-07

**Authors:** 


### HEALTH FOR CHILDREN.

THREE times as many children die in cities as in the country, and half the children born do not reach ten years. Such a result could never have been intended by the wise and kind Maker of us all. A different result must be brought about, by the exercise of the reason which is implanted in all parents, and which, if properly cultivated and practised in the lights of our time, would soon work a wonderful change in infantile mortality.

1. Children should sleep in separate beds, on mattresses of straw or shucks of corn.

2. Require them to go to bed at a regular early hour, and let them have the fullest amount of sleep they can take, allowing them in no case to be waked up.

3. Except a rug beside the bed, there should be no carpet on the floor of their chamber, no bed or window curtains, no clothing of any description hanging about, no furniture beyond a dressing-table and a few chairs, no standing fluids, except a glass of water, and nothing at all in the way of food, or plants or flowers. In short, a chamber should be the cleanest, driest, coolest, lightest and most barren room in the house, in order to secure the utmost purity of air possible.

4. Make it your study to keep your children out of doors every hour possible, from breakfast time until sundown, for every five minutes so spent in joyous play increases the probabilities of a healthful old age.

3. Let them eat at regular hours, and nothing between meals; eating thus, never stint them; let them partake of plain substantial food, until fully satisfied. Multitudes of children are starved into dyspepsia. The last meal of the day should be at least two (2) hours before retiring.

6. Dress children warmly, woollen flannel next their persons during the whole year. By every consideration, protect the extremities well. It is an ignorant barbarism which allows a child to have bare arms, and legs and feet, even in summer. The circulation should be invited to the extremities; warmth does that; cold repels it. It is at the hands and feet we begin to die. Those who have cold hands and feet are never well. *Plenty of warmth, plenty of substantial food and ripe fruits, plenty of sleep, and plenty of joyous out-door exercise, would save millions of children annually.*

